# Culturally tailored, peer-based sleep health education and social support to increase obstructive sleep apnea assessment and treatment adherence among a community sample of blacks: study protocol for a randomized controlled trial

**DOI:** 10.1186/s13063-018-2835-9

**Published:** 2018-09-24

**Authors:** Azizi A. Seixas, Chau Trinh-Shevrin, Joseph Ravenell, Gbenga Ogedegbe, Ferdinand Zizi, Girardin Jean-Louis

**Affiliations:** 1 Department of Population Health, New York School of Medicine, New York, NY USA; 2 Department of Psychiatry, NYU Langone Health, New York, NY 10016 USA

**Keywords:** Obstructive sleep apnea, Peer, Education, Blacks, Home-study

## Abstract

**Background:**

Compared to whites, blacks are at increased risk for obstructive sleep apnea (OSA) yet less likely to adhere to physician-recommended sleep assessment and treatment. Poor OSA health literacy and lack of social support to navigate the current healthcare system are two potential barriers to adequate OSA care. This study is designed to address these barriers by evaluating the effectiveness of a peer-based sleep health education program on adherence to OSA assessment and treatment among blacks at risk for OSA.

**Method/Design:**

In a two-arm, randomized controlled trial, we will ascertain the effectiveness of peer-based sleep health education and social support in increasing OSA evaluation and treatment rates among 398 blacks at low to high OSA risk. Participants at risk of OSA will receive quality controlled, culturally, and linguistically tailored peer education based on Motivational Enhancement principles over a period of 12 months. During this 12-month period, participants are encouraged to participate in a sleep home study to determine risk of OSA and, if found to be at risk, they are invited to undergo a diagnostic sleep assessment at a clinic. Participants who are diagnosed with OSA and who are prescribed continuous positive airway pressure treatment will be encouraged, through peer-based education, to adhere to recommended treatment. Recruitment for the project is ongoing.

**Discussion:**

The use of a culturally tailored sleep health education program, peer health educators trained in sleep health, and home-based sleep assessment are novel approaches in improving OSA assessment and treatment adherence in blacks who are significantly at risk for OSA. Empirical evidence from this trial will provide clinical and population level solutions on how to improve and increase assessment and treatment of OSA among blacks.

**Trial registration:**

NCT02427815. Registered on 20 April 2015.

ClinicalTrials.gov title: Sleep Health Education and Social Support Among Blacks With OSA.

**Electronic supplementary material:**

The online version of this article (10.1186/s13063-018-2835-9) contains supplementary material, which is available to authorized users.

## Background

**Fig. 1:**
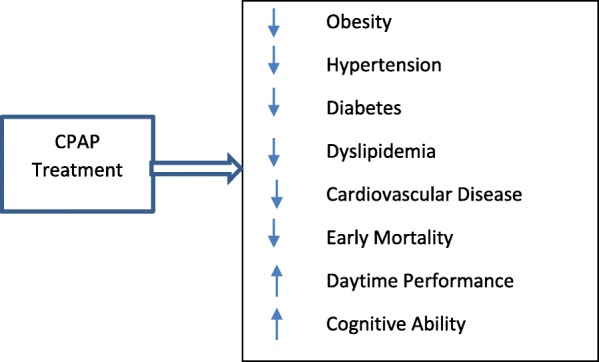
Effects of obstructive sleep apnea treatment on health outcomes

Obstructive sleep apnea (OSA) is a sleep breathing disorder marked by partial (hypopnea) or complete (apnea) blockage of airflow during sleep. Common OSA symptoms include heavy snoring, choking, and gasping for air [1–4]. Untreated OSA can have deleterious health and quality of life consequences for an individual, including, but not limited to, excessive sleepiness, obesity, diabetes, hypertension, stroke, arrhythmia, chronic heart failure, left ventricular failure, atrial fibrillation, tachycardia stroke, road traffic accidents, and mortality [5, 6]. However, OSA-related health consequences are generally preventable and treatable with adequate OSA treatment and management (Fig. 1) [5, 6]. Improving sleep in individuals with OSA may reduce cardiovascular disease (CVD) risk [7], enhance brain function [8, 9], and increase workplace productivity [10]. Studies indicate that continuous positive airway pressure (CPAP) therapy, the gold standard for OSA treatment, is associated with (1) a CVD risk reduction of 64% [7]; (2) reduced brain injury and cognitive dysfunctions [8, 9]; (3) increased workplace productivity [10]; (4) improved hemodynamic functions and metabolic diseases [11–13]; and (5) reduced OSA morbidity [14] and early mortality [15, 16] (Fig. 1).

Although national prevalence data on OSA indicate that all racial/ethnic groups are affected by OSA, some groups are at disproportionate risk. In a population-based study of American adults, aged 40–60 years, OSA was more prevalent among minority groups (16.3%) compared with whites (4.9%) [17–21]. Among black communities, the risk for OSA has ranged from a two- to three-fold risk compared to non-Hispanic whites [18–20]. Though blacks are at elevated risk for OSA, the actual prevalence of OSA among a community sample of urban blacks using self-report and objective assessment is unclear, which is why the proposed study will add significantly to the literature.

Despite racial/ethnic disparities for OSA and OSA-related morbidity, there has been scant attention focused on understanding and addressing racial/ethnic differences in the prevalence of OSA, including the development of targeted OSA screening and treatment interventions [19]. It is unclear whether the nature and extent to which racial/ethnic differences in OSA screening and treatment persist is due, in part, to the lack of public and/or healthcare professional awareness [22–26] or due to poor provider adherence of recommended screening guidelines for OSA. This is further complicated by previous findings that demonstrate that blacks tend to underreport sleep problems [27, 28]. Although the frequency of self-report snoring, a primary OSA symptom, is higher among blacks compared to whites, blacks are less likely to report snoring [29, 30]. Lack of recognition that OSA is a CVD risk factor might also explain why only 38% of black patients with metabolic disease and conditions in community-based clinics adhered to physician-recommended sleep evaluation [31]. To address these barriers, an intervention is needed to increase the rates of OSA assessment and treatment among blacks.

### OSA assessment and treatment barriers among blacks

Unfortunately, blacks have little knowledge of

1. OSA knowledge and its related morbidity;
2. OSA assessment (which includes self-report screens, home studies and clinic studies using polysomnography);
3. Treatment compliance, as compared to whites;

In a previous study [31], only 26% of blacks at risk for OSA (generally based on self-reported screeners, such as a score ≥ 6 on the Apnea Risk Evaluation System (ARES) questionnaire, which was the metric used to determine risk for the current study) adhered to recommended evaluation and treatment. However, of those who adhered to recommended sleep evaluation, 90% received an OSA diagnosis. Additionally, a low percentage of blacks who were at risk for OSA went to a sleep clinic for an OSA diagnosis. These findings highlight the importance of addressing early screening and treatment adherence among blacks with high OSA risk in order to reduce sleep-related health disparities.

Our unpublished focus-group findings of community members indicate that peer health educators, representative of the black community, might yield greater compliance in OSA assessment and treatment compared to a health professional. Based on these recommendations, we included community-based peer health educators to deliver tailored sleep health education to increase OSA assessment and treatment compliance. In addition, participants suggested that a home-based sleep study might yield greater compliance in OSA assessment (self-report screener, home-study, and clinic study using polysomnography).

In sum, the current study’s inclusion of community-based peer health educators and home-based studies is an outgrowth of previous studies indicating that poor adherence to CPAP (< 4 hour a night for 70% of the nights) is a significant barrier to successful management of OSA [32]. Compared with whites, blacks have less frequent contact with the healthcare system partly because of mistrust or negative past experiences, as well as not always perceiving the need for regular clinic visits [33–36]. Our use of peer health educators from the community and a home-based study are attempts to overcome the aforementioned barriers and increase the facilitators of OSA assessment and treatment, as well as to destigmatize these and demystify the healthcare system.

### Study rationale

The primary aim of this trial is to ascertain (1) the effectiveness of tailored, peer-based sleep health education and social support in increasing adherence rates to recommended OSA evaluation (adherence to sleep home-based study is categorized as ‘yes’ or ‘no’ depending on whether the individual participated in a sleep home-based study and adherence to sleep clinic assessment is categorized as ‘yes’ or ‘no’ depending on whether the individual had a laboratory-based sleep clinic assessment) and treatment among blacks at risk for OSA (adherence will be defined as whether the participant wore their CPAP device > 4 hours a night for 70% of the nights); and (2) the rate of OSA among black men and women at the community level using home-based sleep recordings. Early identification of OSA via home-based studies in this high-risk population could potentially lead to early OSA treatment and prevention and may reduce subsequent health consequences such as CVD and premature death. Our study can be used as a national model for how to increase racial/ethnic minority engagement in the healthcare system through culturally responsive strategies that bridge underserved communities. Thus, our proposal to screen blacks in community settings, such as barbershops, beauty salons, and places of worship, will maximize the likelihood of recruiting and enrolling a representative sample of blacks.

## Methods and design

In a two-arm, randomized controlled trial, we will ascertain effectiveness of peer-based sleep health education and social support in increasing OSA evaluation and treatment rates among 398 blacks at risk for OSA (198 participants in treatment group and 198 participants in attention-control group), over a 12 month period. Participants in the intervention arm will receive quality-controlled, culturally, and linguistically tailored peer education using a sleep health education manual developed based on principles of motivational enhancement. Participants in the treatment arm will each receive up to five peer-based sessions promoting OSA evaluation. Those with an OSA diagnosis will receive five additional peer-based sessions promoting OSA treatment compliance and adherence from a peer health educator contingent upon poor treatment adherence from CPAP-based reports. Those in the attention-control arm will receive standard OSA literature from the study staff. Regardless of group assignment, we will ascertain all outcomes at baseline and at 6- and 12-month follow-up assessments.

**Fig. 2:**
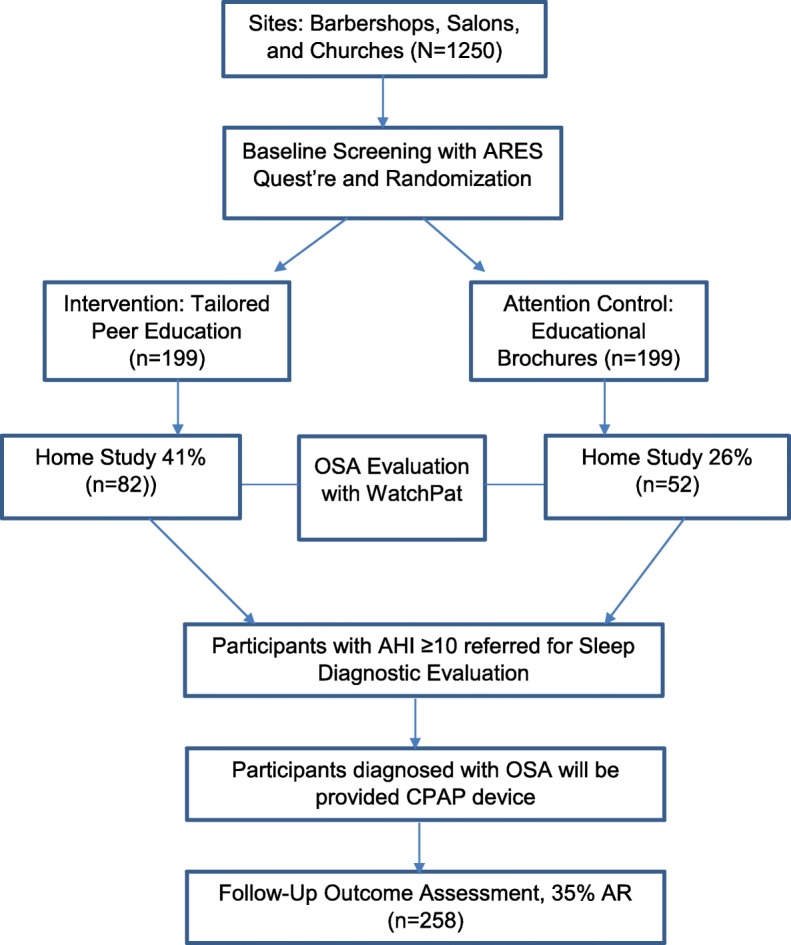
Theoretical framework for the impact of peer-based behavioral intervention on adherence to recommended obstructive sleep apnea evaluation and treatment

**Fig. 3:**
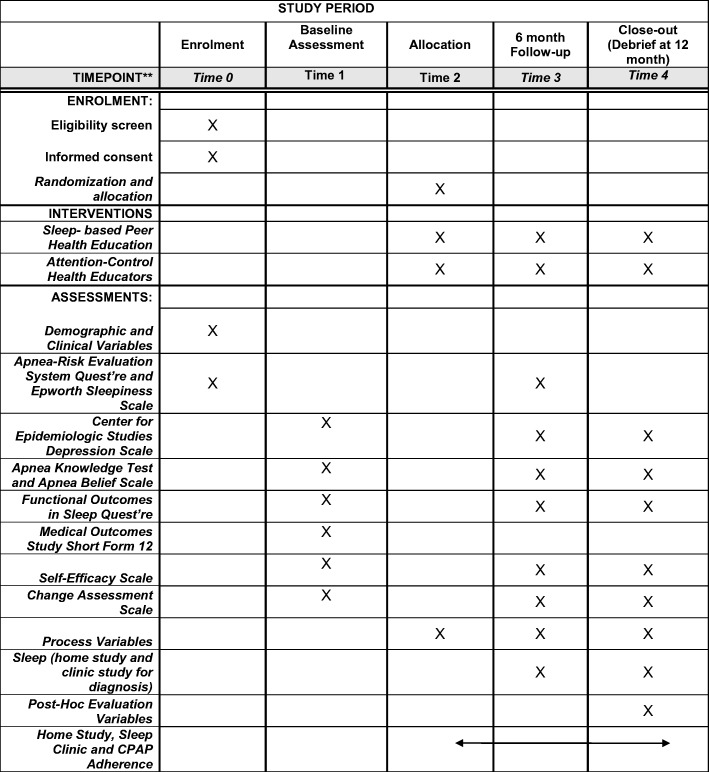
Study design

The study was approved by the New York University Langone Health Institutional Review Board and all participants will be consented by study staff before enrollment. The trial is in accordance to the Consolidated Standards of Reporting Trials (CONSORT) Statement on randomized trials for non-pharmacological treatment and the Standard Protocol Items: Recommendations for Interventional Trials SPIRIT checklist (see Additional file 1 for CONSORT Figure, SPIRIT Figure and checklist Figs. 2 and 3) [37, 38].

### Theoretical framework of intervention

**Fig. 4:**
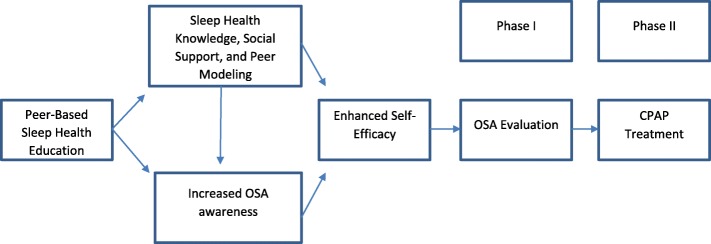
SPIRIT checklist

The theoretical framework (Fig. 4) for the study was adapted from the Centers for Disease Control AIDS Community Demonstration Projects [37, 38], using the following elements: (1) in-depth community assessment to articulate project aims, (2) focus on making risk reduction socially desirable, (3) mobilization of credible peers (or members of their community) to dispense sleep health education, (4) use of role modeling as the primary medium for health messaging, and (5) utilization of a medical device that can be used in a non-threatening way at home. The study also draws from Rothman and Tropman’s Model of Community Organization viewing change as a process seeking participation of a broad cross-section of the community, attempting to identify and solve its own challenges [39, 40]. Using barbershops, salons, places of worship, and community-based organizations as a social network, will increase empowerment, participation, and competence of stakeholders by focusing on (1) participatory training of peer educators, (2) distribution of tailored sleep health materials, and (3) referrals and treatment of blacks meeting OSA diagnostic criteria.

The trial will be conducted in five phases, which we describe below. In Phase I, we will train health educators both in the intervention and control groups. In Phase II, we will recruit and screen a community sample of blacks. In Phase III, peer health educators will provide counseling to eligible participants (blacks at risk for OSA) in order to have them adhere to an OSA home-based assessment and diagnostic test to determine treatment (in most cases CPAP). In Phase IV, peer health educators will provide more counseling to participants diagnosed with OSA and CPAP in order to optimize participant adherence to recommended treatment. Finally, in Phase V, the study team will debrief enrolled participants.

### Phase I: peer health educator training (intervention)

Much of the literature addressing the underutilization of health services among racial/ethnic groups points to lack of access to care [41]. Reduced access is in part attributed to a general mistrust of the healthcare system [42–45]. Individuals from racial/ethnic minority communities are less likely to seek medical help for their ill health [46], preferring to consult with an elder family member, a pastor, or a folk practitioner to deal with health-related issues [47]. Minority patients who are activated in the healthcare system report better patient satisfaction, trust building, active participation in clinical encounters, and more effective communication when practitioners are of similar racial/ethnic heritage [48]. This highlights the need for a caring and trust-building approach in addressing barriers preventing recommended evaluation practices [49, 50]. Several intervention strategies (e.g., peer education) have specifically built upon the importance of a caring and trust-building approach. The key element is that the intended audience trusts and respects them.

Peer health educators must be intimately familiar with the social sanctions, rituals, values, and rules of conduct within the community, which may have direct bearing upon intended intervention goals [51–56]. Peer health education refers to the teaching or sharing of health information, values, and behaviors by members of similar sociodemographic status [57]. It is a growing modality for support, encouragement, follow-up, and assistance tailoring care plans [58] and serves several functions, including (1) bridging the gap between communities and the healthcare system, (2) promoting wellness by providing culturally appropriate health information to clients and providers, (3) assisting in navigating the healthcare system, (4) advocating for individual and community needs, (5) providing direct services, and (6) building individual and community capacity. Success of health education in racial/ethnic minorities is achievable only if it is provided by peer educators, recruited directly from the partner community and trained to accomplish the following tasks [59]: (1) help increase access to health services for individuals at greatest risk for morbidity and mortality, (2) improve quality of care, and (3) reduce healthcare costs.

#### Training of peer educators (intervention arm)

Barbers, stylists, and leaders in faith-based communities are influential peers who are uniquely positioned to administer the proposed health education program [60, 61]. With adequate training and supervision, we expect that they will be able to utilize motivational enhancement techniques (the trans-theoretical change model) leading to the desired behavior, namely seeking OSA evaluation and adhering to treatment. These techniques have proven effective in a similar project that aimed to improve blood pressure control among blacks in community settings [62, 63]. As indicated in Fig. 4, the conceptual framework for training educators is modeled after Behavior Change Communication best practices and recommendations [64, 65]. Peer health educators are taught how to deliver tailored motivational sessions depending on where an individual is on the Stages of Change model (e.g., pre-contemplation, contemplation, preparation, action, maintenance, relapse prevention), which will be determined by baseline survey on Stages of Change [66]. For our current study, health educators will apply for the health educator job and will then be interviewed by the project manager and coordinator. To be considered for the peer health educator position, individuals have to meet the following qualifications: (1) previous experience working/volunteering with programs or organizations that tackle health or social issues in black communities; (2) knowledge of community resources and organizations in Brooklyn, Bronx, Queens, and/or Staten Island; (3) available to work evenings and weekends regularly; (4) ability to juggle priorities and manage time productively; (5) effective oral and written communication skills; (6) energetic open-minded people-person; (7) flexible, ability to change gears at a moment’s notice and ‘go with the flow’; (8) thrives in a fast-paced environment; (9) highly organized, pays attention to detail, and can follow strict protocols; (10) creative problem-solver; (11) comfortable using a computer (Word, Excel, Access, Outlook); and (12) a minimum of a high school education or GED. Peer health educators will not be required to have previous training or expertise in medicine or health training. Health educators will have a maximum case load of 3–4 participants at a given time and will provide support and counseling for each participant for 10 sessions.

#### Sleep health manual for peer educators

A sleep health training manual was developed for health educators in the intervention arm. The manual delineates the mode of delivery, details on objectives for each encounter, and the ideal and minimally acceptable intervention ‘dose’. The peer health educator will utilize a graded dosage based on the participant’s educational and motivation levels to seek OSA evaluation. For example, a peer health educator will provide more introductory messages about what is OSA to someone who is in the pre-contemplative phase, while the information and support they provide a participant in the action phase will be qualitatively different as they may be more likely to agree to OSA evaluation compared to someone at the pre-contemplative or contemplative phases. Generally, in the first encounter, the peer health educator begins with rapport building, exchanging pleasantries, inquiring about the participant’s welfare, and then starting with a discussion of general problems while gradually introducing sleep health messages. The peer health educator will also discuss benefits of reducing OSA health risk behavior (e.g., smoking, drinking, sedentary lifestyle and poor diet). In the second encounter, the educator reinforces the importance of OSA evaluation and overcoming barriers and inquires if the participant has had a home visit. In the second encounter the peer health educator addresses decisional conflicts and encourages the participant to make an appointment for OSA evaluation. In subsequent encounters, the peer health educator inquiries about the general welfare and health status of the participant, reiterates health messages, and inquires whether the participant has had an OSA evaluation and/or has received treatment. The manual also enumerates strategies for overcoming challenges, specifies referral procedures, and describes strategies to check participants’ understanding of information and the mechanisms in place for monitoring fidelity.

Training to support and monitor fidelity to the intervention will be conducted in a standardized manner to (1) ensure that it is similar for all peer health educators and to ensure adherence to intervention protocol, (2) ensure educator’s skill acquisition using well-defined criteria (they will be certified before the intervention begins and periodically), (3) minimize drift in skills by providing booster sessions through both in vivo observations and review of behavioral checklists, and (4) accommodate differences in skill levels, experience, and professional background of educators.

#### Treatment fidelity

Peer health educators will attend six training sessions on the basics of sleep science, motivational interviewing, ethics in research, and assessment and treatment of OSA. During these meetings, the importance of and rationale for the study as well as best practices of health education (such as providing accurate information about sleep and social support to participants) will be reinforced. In order to become an official peer health educator, individuals will have to successfully pass a scenario- and practice-based assessment. Peer health educators will be evaluated on the following competencies: (1) mastery of OSA and sleep health information, (2) Readiness to Change model, and (3) interpersonal skills (clear and effective communication, cultural sensitivity, ability to create positive and effective rapport) by a sleep health trainer and two sleep health specialists. Quarterly booster sessions will be held to reinforce treatment fidelity, address any project-related problems they may be encountering, and to foster self-confidence, self-efficacy, and enthusiasm for the study. Participants in the intervention arm will receive 10 counseling sessions from their matched health educator. Additional medical counseling may be provided to participants by the treating sleep physician or healthcare provider (who does not belong to the study) at the participants’ request.

#### Study setting: study sites/population

Barbershops, salons, places of worship, and community-based organizations are ideal places to recruit hard-to-reach research participants such as racial/ethnic minorities*.* Evidence shows that a grassroots, community-centered approach using institutions that are well accepted, providing culturally and linguistically tailored health information, is the most effective way to reach racial/ethnic minorities in medically underserved areas [60, 67]. Barbershops, salons, and churches are ideal venues to communicate important health messages to blacks because of their established credibility as forums of in-depth discussions, information gathering, and sharing of personal experiences [60, 68, 69]. Thus, our intervention will use these establishments to meet the study’s aims. We will recruit from a wide network of barbershops, salons, places of worship, and community-based organizations.

We expect trained peer health educators will be influential in changing attitudes toward OSA evaluation because they have a high degree of homophily with their assigned participant [70, 71]. The theory of innovation suggests that interpersonal influence is the most effective way to change attitudes and share skills [71]. The ubiquity of racial/ethnic minority barbershops, beauty salons, places of worship, and community organizations across the United States [72] (credible institutions to communicate health messages to blacks), and the high degree of homophily (based on similarity in race, gender, beliefs, values, education, and social status) between peer educators and participants increases the likelihood of providing widespread OSA education across diverse groups.

#### Selection of study sites

Barbershops, salons, and churches will be selected based on established criteria, namely visibility, accessibility, cleanliness, safety, longevity, and cooperativeness [59]; preference will be accorded to those providing private meeting space for confidential interactions between educators and participants. Recruitment procedures will be discussed with site owners before scheduled visits. Respective owners will introduce the recruitment team, who will then approach potential participants (while waiting at the shop/salon or after church services) to describe the protocol. Participating recruitment sites are compensated US$ 250 annually. As in our previous study [73], staff will begin by exchanging pleasantries with potential participants followed by an introduction of study rationale. If interest is expressed, eligibility will be confirmed and consent obtained.

### Phase II: recruitment, screening, randomization, and survey administration

#### Recruitment and screening

The sample will include blacks recruited from barbershops, salons, and faith-based organizations in New York City. We intend to screen approximately 1250 blacks and expect that approximately 398 will be enrolled in the study (Figs. 2 and 3). Research assistants will screen potential participants to determine eligibility on a wide range of exclusion and inclusion criteria at community-based settings such as faith-based organizations, barbershops, beauty salons, health fairs, and community-based organizations. If participants are eligible, research assistants will provide informed consent (Additional file 1). Participants will be re-consented if procedures of study significantly change.

##### Eligibility criteria (inclusion and exclusion)

Eligible participants will (1) self-report their race/ethnicity as black, African American, or African ancestry; (2) be ≥ 18 years old; (3) accessible by telephone or cellphone; (4) have no plans to move away from the region within the year following enrollment; (5) consent to participate in the study, which includes permission to release medical record information; and (6) have a positive screening for OSA, based on whether an individual receives a score ≥ 6 on the ARES Questionnaire. Participants are ineligible if they (1) are involved in another sleep study; (2) know someone who is participating in this study; (3) plan to move away in the next year; (4) had a heart attack or stroke within the past 12 weeks; (5) are pregnant; (6) refuse to use the ActiGraph activity monitor; (7) are non-English speaking; or (8) refuse to use the WatchPAT™ home sleep test device. Participants who are currently being treated for sleep apnea will not be eligible for the study. We do not anticipate that concomitant treatment for other medical conditions will preclude participation in the study.

#### Survey data collection

##### Measures at baseline, 6-month and 12-month follow-up

Participants will complete the ARES™ [74], Center for Epidemiologic Studies Depression Scale [75, 76], Apnea Knowledge Test [77], Functional Outcomes Sleep Questionnaire [78], Medical Outcomes Study Short Form [79, 80], Self-Efficacy Scale [81], and Change Assessment Scale [82] (Fig. 3, Additional file 2).

##### Sociodemographic variables

Sociodemographic variables will include age, type of health insurance, marital status, education, place of birth (for example, US vs. Caribbean), years in current residence and locality, number of children living in household, number of adults living in household, self-described ethnicity, and annual household income.

##### Epworth sleepiness scale

This questionnaire uses a Likert-type scale in which the respondent indicates the most appropriate number ranging from 0, would never doze or sleep, to 3, high chance of dozing or sleeping, based on a given situation. A score of 10 or more is considered excessive sleepiness [83].

##### Apnea Knowledge Test (AKT) and Apnea Beliefs Scale (ABS)

These two questionnaires assess participants’ understanding and beliefs of OSA and CPAP treatment. The AKT includes 13 questions with a yes/no response choice and 2 open-ended questions; the ABS includes 24 items on a 5-point Likert scale ranging from ‘strongly agree’ to ‘strongly disagree’. A higher score on the AKT indicates better knowledge and understanding of the illness and treatment and a higher score on the ABS indicates greater likelihood of adherence to treatment [84].

##### Change assessment scale

This is a 32-item instrument that assesses a person’s readiness to change. Participants evaluate the extent to which they strongly agree or strongly disagree with various statements such as “I’ve been thinking that I might want to change something about myself” and “I have problems and I really think I should work on them” [85].

##### Medical history

Medical history includes family history of sleep disorders, obesity, diabetes mellitus, hypertension, dyslipidemia, and CVD, history of other preventive behaviors (for example, prostate exams), and other health behaviors (for example, regularity of physical examinations, cholesterol screening, smoking and drinking habits, and exercise habits).

##### Apnea-Risk Evaluation System (ARES) questionnaire

We will use the ARES to identify individuals who are at high OSA risk. This tool includes questions on sleep patterns, daytime functioning, knowledge of sleep apnea, and diseases associated with sleep apnea (that is, hypertension, diabetes mellitus, heart disease) [86]. Other data solicited in the ARES include sociodemographic information, medical comorbidities, the Epworth Sleepiness Scale, and frequency of breathing abnormalities. The questionnaire has a sensitivity of 0.94, specificity of 0.79 (based on a clinical cut-off of apnea-hypopnea index (AHI) > 5), positive predictive value of 0.91, and negative predictive value of 0.86 [87]. Our rationale for using the ARES questionnaire in a diverse sample is also based on findings in our previous studies [73, 88, 89], which indicated that the ARES accurately detected OSA risk among black participants. Other evaluation tools, such as the Berlin, the American Society of Anesthesiologists checklist, or STOP-BANG, have not been specifically tested among blacks at the community level.

#### Data collection and management

Once baseline data is obtained, contact information of consenting participants will be provided to the data manager, who will maintain a registry of enrolled participants (control group and intervention group). This registry will be used to track them during the study period. Using computer generated schedules; the data manager will perform blind outcome ascertainment at 6 and at 12 months.

The data manager will perform interim analysis to ensure that selection and attrition biases do not occur if refusal occurs after recruitment or randomization. Selection bias might result from participant’s refusal to participate and attrition bias could result in an unbalanced design. Appropriate statistical techniques, such as Heckman 2-stage approach (Generalized Estimation Equations (GEE) Probit Model) [90] and Monte Carlo (EM algorithm) [91] analysis, will be used to address such biases or problems that might result from missing data. Adequate statistical corrections will be made in final statistical analyses to increase the generalizability of findings. Sociodemographic data obtained from those who did not participate will be used to determine whether individuals who did not participate are similar to those in the study (selection bias). Likewise, baseline characteristics of those who withdrew from the study will be contrasted with completers to determine whether any attrition bias may have occurred. Site-specific effects (shops vs. salons vs. faith-based site) will also be examined. If these analyses reveal meaningful between-group differences, such characteristics will be treated as covariates. If we identify missing data in the analysis (e.g., logs, checklists, rapport), we will use Kelsey’s method strategies to remedy missing data [92]. Although we do not plan to perform extensive cost-benefit analysis, we will document implementation costs (e.g., personnel, training costs, home visits, and postage and handling). In addition to performing analytical tests to minimize the potential confounding effects of missing data, we will engage in evidenced-based research design (recruitment and retention) strategies to minimize the possibility of missing data, some of which include (1) closely monitoring research milestones and targets and ensuring that participants reach these with the help of a health educator with whom the participants have a good rapport; (2) limiting the amount of research burden; (3) keeping close contact with participants through the project coordinator or health educator; and (4) providing adequate and fair monetary incentives for participating in the research study.

#### Randomization to intervention or attention-control groups: allocation and blinding

Participants will be randomly assigned to either the intervention arm (partnered with a peer health educator who received the culturally and linguistically tailored sleep health education and was trained for 8 weeks) or the attention-control group (the comparative group where participants are partnered with a peer health educator who did not receive tailored sleep health education but only standard health training for a 1 hour session). The unit of randomization is the participant and thus the unit of analysis will be at the participant level. All study staff except for the data manager and project coordinator will be blinded to participant assignment. The data manager generates the randomization sequence for each eligible participant and the study coordinator randomly assigns peer health educators to participants assigned to treatment and attention-control groups. We will un-blind participant assignment only in cases where the participant’s health is at risk due to CPAP usage (Fig. 3).

##### Intervention group

Participants in the intervention arm of the study will receive counseling from peer health educators who received culturally and linguistically tailored sleep health education. The details and contents of peer health educator training are described in the Peer Health Educator Training section above. Participants will receive up to 10 sessions from their health educator with the hopes of motivating the participant along the OSA continuum of care, which entails screening, home-study assessment, sleep clinic assessment, diagnosis, treatment customization, and treatment adherence.

##### Attention-control group

Participants in the attention-control group will receive standard sleep literature by a control group peer health educator, who will provide general information about OSA and access to available sleep services through pamphlets. Similar to procedures that will be followed for participants in the intervention arm, health educators will advise all participants at risk for OSA to seek OSA home evaluation by contacting the research program coordinator to schedule a home visit and move through the continuum of care described above.

### Phase III: OSA assessment and diagnosis – home study and sleep clinic assessments and treatment customization

#### Primary trial outcome: sleep home study

The research staff will conduct an 8-day home study to collect sleep data via actigraphy (ActiGraph WGT3X-BT monitor) and a daily sleep diary, as well as environmental influences of sleep, such as noise and temperature, two factors that may confound sleep duration and quality. On day 1 of home sleep, a study technician will visit the participant’s home to place the ActiGraph watch on the participant’s wrist as well as connect a sound device and thermometer to capture the participant’s physical sleep environment. During the visit at the participant’s home, staff will demonstrate how the WatchPAT™ home sleep test device is used, worn on the wrist and fingertip on the last night of a 7-day sleep diary. On the seventh day of the home evaluation, the study’s technician will re-visit the participant’s home to set-up a device, the WatchPAT™ portable sleep test device to determine the severity of OSA risk. The WatchPAT™ is an in-home device utilized for OSA evaluation cleared by the Food and Drug Administration and approved by the Center for Medicare & Medicaid Services [93, 94]. It measures blood oxygen saturation, pulse rate, airflow, respiratory effort (a combined signal using pressure transducer sensing forehead venous pressure, venous volume by photoplethysmography), and snoring levels. Automated analysis of physiologic data yields OSA indices that reflect OSA risk, which is verified by a trained human scorer to ensure accuracy of signal interpretation. The device integrates physiologic, clinical, and anthropomorphic data to determine the presence and severity of OSA. Correlation between the WatchPAT 200 and PSG recordings was 0.96 using AHI with a 4% desaturation. Diagnostic sensitivity and specificity of in-home WatchPAT using AHI ≥ 15 (in the absence of OSA symptoms) are 0.85 and 0.91, respectively [93]. Based on Medicare guidelines [95], those with an AHI ≥ 10 will be referred for treatment (American Academy of Sleep Medicine guidelines) [96]. We chose this criterion to ensure that we reduce the rate of false negative results. In the 7-day sleep diary, participants will indicate (1) nap time and duration, (2) medications/drugs promoting night-time sleep or daytime alertness (e.g., caffeine), (3) time to bed, (4) time of lights off, (5) final time of awakening, (6) final time of arising, and estimated (7) sleep latency, (8) sleep duration, (9) wake after sleep onset, (10) sleep quality, and (11) daytime sleepiness. On day 8, the study staff will retrieve all devices and data from the sleep diary.

After the home sleep study, the study staff will provide the results to the study physician to determine the risk of sleep apnea from the WatchPAT™ home sleep recorder device. The study physician will discuss the home study results with all participants and will recommend treatment to those meeting OSA diagnostic criteria (AHI > 10). Participants who do not meet OSA diagnostic criteria will be transferred to the project coordinator either for study close-out since they no longer need to continue their participation in the study, for future clinic-based diagnostic assessment, or for treatment customization. Participants who are at risk for OSA will be referred for further diagnostic assessment and treatment to a sleep clinic accredited by the American Academy of Sleep Medicine. Expenses incurred from laboratory and CPAP procedures recommended by sleep clinicians in our catchment area will be billed directly to the participant’s insurance. In the rare instance that a participant might fail to obtain insurance, the study project will absorb some medical costs and a CPAP device will be dispensed as an in-kind contribution.

#### Secondary trial outcome: sleep clinic assessment, diagnosis, and treatment customization

Participants who have a high AHI (≥ 10) will be referred for sleep assessment to determine OSA diagnosis and appropriate treatment. Peer health educators will support participants as they navigate this phase of their care by helping them with scheduling appointments and occasionally accompanying them to appointments if requested by the participant, as well as providing general information about the sleep assessment. The purpose of this level of social support is to demystify the OSA assessment (which includes self-report screener, home study, and clinic study using polysomnography) experience, which can be daunting and might deter participants from undergoing the sleep assessment. In our previous research, we found that blacks were less likely to adhere to recommended OSA treatment and therefore the use of peer health educators is a practical way of increasing CPAP adherence.

### Phase IV: third trial outcome – continuous positive airway pressure (CPAP) adherence

Participants who are diagnosed with OSA and are prescribed CPAP will be provided a CPAP machine at their residence by a technician from the CPAP company. The technician will provide standard initialization of the CPAP machine and a to-do kit that provides general use and care guidelines for the machine. The participant’s assigned peer health educator will resume peer education sessions with the aim of motivating the participant to adhere to the recommended use of the CPAP machine. This is a critical period in OSA treatment, as a significant number of patients do not adhere to recommended treatment. Therefore, the role of the peer health educators is very important because they can provide tailored sleep health education on the importance of adhering to CPAP, address barriers preventing the participant from complying with treatment, and help to motivate participants to adhere to treatment.

### Phase V: debriefing and exit interviews

#### Exit interviews

At the end of the 12 month enrollment in the study, all participants will be debriefed by the project coordinator. Participants will complete the same baseline questionnaires and additional questionnaires designed to understand why participants adhered to the intervention and how the intervention might be refined to be more effective.

#### Compensation

Participants can receive up to US$ 125 for completing all the study visits, including US$ 5 for the screening visit, US$ 60 for the baseline and home study visit, US$ 30 for the 6-month follow-up visit, and US$ 30 for the 12-month follow-up visit. If participants choose to withdraw from the study before finishing the study*,* they will be paid up to the point of withdrawal.

#### Data safety and monitoring

All participants’ data and patient health information will be confidential and protected during and after the trial. A code will be used to identify study participants, which will not be given to anyone outside of the study staff unless required by law. All paper records are stored in an area with two locked doors and/or drawers. Only the study staff will have access to the locks. All computerized records are password protected available only to the study team. We will use a data monitoring committee independent of the funding source to provide oversight on integrity of the collected data. We will perform yearly audits to ensure that study protocol is followed.

#### Risk/benefit assessment

Sensors used for the sleep study recordings may cause minor skin irritation among people with sensitive skin; this does not usually require medical attention. Such irritation can easily be resolved with over-the-counter ointment that can be obtained at a local drug store. Participants may not directly benefit from being in this study. However, possible future benefits include increased awareness and learning about sleep apnea and receiving treatment for sleep apnea among those with an OSA diagnosis. If participants experience any harm due to participating in the study, we will follow all New York University ethical review board guidelines, which include (1) ensuring medical safety of participants by providing any medical treatment needed; (2) investigating the incident, determining if risk is inherent to study procedures, and submitting an adverse event report to the New York University ethical review board; and (3) making necessary updates to the protocol and notifying currently enrolled participants about the given risk.

#### Ethics and dissemination

The current trial and aforementioned procedures were approved by the New York University School of Medicine Institutional Review Board. Any changes to the protocol will need to be approved by the Institutional Review Board and the enrolled participants and funder will be notified. At the completion of data collection, the principal investigator and data analyst will process and analyze the data. Findings will be disseminated to the communities from which participants were recruited as well as to the funder. The data will be made publicly available 5 years after completion.

#### Preliminary findings

We conducted a feasibility study using OSA screening tools among blacks at the community level to provide (1) estimates of the likely rates of OSA in the community, which will in turn guide our recruitment strategies, (2) an empirical basis for our sample size calculations, and (3) preliminary evidence that education and support offered by a peer health educator led to OSA evaluation among blacks at risk.

Over a 15-day period, we randomly assigned 27 blacks to an intervention group (peer health educator) and 25 to a control group (publicly available sleep brochures and literature). The desired behavior was limited to ‘making an appointment for an OSA assessment’. Analysis showed that 26% of blacks in the control condition made an appointment for OSA assessment, whereas 41% of those exposed to the intervention arm made an appointment. Although results were in the expected direction, we were unable to determine whether participants would show up for OSA assessment and/or adhere to treatment. This requires long-term monitoring and justifies the purpose of our study.

#### Sample size and power analysis

We plan to screen approximately 1250 blacks in the community. Assuming 39% of blacks are at risk for OSA, we propose to enroll 398 men and women (199 per study arm). With a 35% attrition rate, 258 will have provided adherence data during 6-month follow-up assessment. Based on the pilot data, we expect that 41% in the intervention arm (*n* = 82) and 26% in the attention-control arm (*n* = 52) will have completed all aspects of the trial, including site interview, home evaluation, and follow-up assessment (Fig. 2).

The primary study outcome is receipt of home OSA evaluation after tailored peer-based sleep health education. Data obtained from a sample of blacks provided a current estimate of the likely OSA evaluation rate we expect in the partner community. Analysis showed that 41% of participants adhered to recommended OSA evaluation after intervention exposure, whereas 26% of participants in a control group agreed to undergo evaluation. These preliminary findings represent the likely adherence rates expected in our proposed study.

We propose to achieve an evaluation rate in our intervention arm of 41% based our pilot study mentioned above. This would represent a 63% greater adherence rate compared with controls, which potentially would have a significant public health impact. Assuming a 15% increase in the likelihood of undergoing home-based OSA evaluation, a standard error of 0.09% and a sample size of 398, the study will be adequately powered (> 85%) to detect a significant increase in adherence. Specifically, an observed difference of 15% would be reported with a 95% CI 7–24%. A two-tailed test, with 80% power and α = 0.035 comparing adherence rates of 26% with 41% requires 199 participants per group. Of note, our sample size estimate adjusted for potential intra-cluster coefficient that may be observed in both the treatment and control arms, which according to our initial pilot was 0.014. The resulting effective sample size (*ESS = [M x K/DE]*) after adjustment for design effect (*DE*)… [*DE = 1 + (n-1)ρ*] was *n* = 398 (199 participants per arm) [97, 98]. The study is also powered to assess differences in adherence rates to CPAP treatment between the two arms considering the likely attrition rate of 35% during follow-up assessment.

#### Statistical analysis plan

To describe sample characteristics, we will use measures of central tendencies and dispersion for continuous data; frequency distributions will be used for categorical data. We will use χ^2^ tests to compare categorical data and GEE to examine relationships between predictors of adherence to OSA evaluation and/or treatment. The main dependent measure will be adherence to OSA evaluation (yes vs. no) and adherence to CPAP treatment (yes vs. no). Effects of sociodemographic and clinical factors will be adjusted. MANCOVA will be used to assess differences between baseline and 6- and 12-month subjective measures (e.g., self-efficacy, apnea knowledge, functional outcomes in sleep). Structural Equation Modeling will be used to examine the path toward adherence to OSA evaluation and/or CPAP treatment, considering the contributions of candidate factors.

##### Primary aim and outcome

To ascertain effectiveness of tailored, peer-based sleep health education and social support in increasing adherence rates to recommended OSA evaluation and treatment among blacks at risk for OSA. We hypothesize that greater adherence will be achieved, defined as successful completion of home sleep study and sleep clinic assessment.

##### Analysis

We will perform analysis on an intention-to-treat basis. We will assume participants in the intervention arm to have received the full intervention dose and those in the control arm to have received no tailored education. Prior to testing for intervention effects, we will verify comparability of the intervention and control arms with respect to all available sociodemographic data using *t* tests and χ^2^ tests. GEE methods will be used to estimate the odds associated with receipt of OSA evaluation contrasting the two study groups. GEE is preferred over standard logistic regression, as it adjusts for clustering effects. Delays in receipt of endpoint (OSA evaluation or treatment) will be adjusted in the models. Since the type of health educator may influence our findings, we will perform statistical analyses that will detect any potential confounding effect that health educator assignment may have on outcomes. This type of method is distinguishable from a traditional cluster randomized trial as the unit of randomization is at the individual/participant level and not groups of individuals.

##### Secondary aim

To ascertain how many among black men and women have OSA or are at risk for OSA at the community level using home-based sleep recordings in accordance with criteria from the American Academy of Sleep Medicine. We hypothesize greater adherence to OSA treatment using telemetry (CPAP use; > 4 hours a night for 70% of the nights) [99–101].

##### Analysis

The analysis plan is similar as for hypothesis 1. We will use GEE to estimate odds of adhering to CPAP treatment at 6 and 12 months. This aim does not require any changes or additions to our procedures.

##### Tertiary aim

To determine which individual- and contextual-level factors affect the relationship between peer health education and OSA evaluation and treatment. We hypothesize that individual-level factors (OSA knowledge, OSA self-efficacy, and past evaluation behavior) and contextual level factors (i.e., noise levels, room temperature, neighborhood factors, trust/rapport with peer educators, family network, and socioeconomic position) will mediate effects of peer education on adherence to OSA evaluation and recommended treatment.

##### Analysis

We chose these individual and contextual factors based on research showing acceptable effect size in predicting adherence status (e.g., home-study adherence, sleep clinic adherence, and CPAP device adherence) [102]. We will examine the path toward adherence to OSA evaluation and CPAP treatment using structural equation modeling, which will determine influences of mediating/moderating factors on the role of peer education in improving adherence to OSA evaluation and treatment. Specifically, we will utilize moderated path analysis using the Baron and Kenny model [97, 98, 103]. Alternatively, we will explore whether causal mediation analysis might prove a better fit for our data than the Baron and Kenny model. Causal mediation analysis compared to structural equation modeling is a more flexible model, as it is based on a counterfactual framework and may be more appropriate for causal inference, especially in the presence of measured confounding [104, 105]. It should be noted that, if our statistical analysis plan changes, we will update this in the *Trials* journal.

#### Ancillary studies and post-trial methods

It is likely that participants from the current study may be eligible for related ancillary studies. In such cases, only participants who endorsed being contacted for future studies will be contacted about ancillary studies. The study team will determine whether involvement in an ancillary study will interfere with the current study. If the ancillary study will interfere, we will defer enrollment until the participant is finished with all study procedures of the parent study. It is also likely that participants may be contacted about future studies after participating in the study. We will only contact participants who requested to be contacted.

## Discussion

The current study is an outgrowth of our previous National Institute on Minority Health and Health Disparities-funded randomized controlled study (NCT01946659), which investigated whether blacks with metabolic syndrome are at increased risk for OSA. During the study, many black patients were unwilling to undergo a laboratory sleep evaluation to determine if they had diagnosable OSA. The proposed study will be the first randomized trial to utilize home-based sleep recordings to assess OSA risk among blacks. In addition, our study is innovative in the following ways.

To our knowledge, this will be the first culturally and linguistically tailored, community-engaged, peer-based sleep health intervention to increase adherence to OSA evaluation and treatment. Although peer education is not a novel concept [60, 106, 107], its effectiveness remains untested among blacks with OSA. Second, while peer education may be effective in increasing OSA assessment behavior, it may be insufficient to achieve optimal adherence to CPAP therapy since common side effects (e.g., mask fitting, pressure, nasal irritation, and claustrophobia) that negatively affect adherence are routinely addressed at the clinic. Thus, individuals showing poor adherence according to web-based CPAP data will receive telephonic interventions from a trained OSA Navigator interfacing with the Project Coordinator. Third, traditional diagnosis of OSA is rendered based on a sleep laboratory assessment, limiting access to many blacks. Fourth, our intervention strategy will be tailored based on information gleaned from focus groups with patients in our previous randomized controlled trial and will draw from family networks, with a focus on input from participants’ partners. Recent evidence shows that partners have either a positive or negative influence on CPAP acceptance [108–111]. Evidence from our previous RCT suggested that partners are instrumental in facilitating adaptation of CPAP therapy in the home environment. Fifth, we will examine both individual- and contextual-level mediators/moderators of intervention effects. Our approach recognizes the interplay of biological and behavioral factors, as well as the influence of contextual factors in determining sleep health behavior in the partner community (Fig. 2) [112].

Our analysis of the BRFSS data revealed several significant social/cultural determinants of sleep deficiency among blacks [113]. They include working more than 40 hours (OR 1.72), caregiving to family/friends (OR 1.23), and lack of emotional support (OR 1.21). Our research among blacks in Brooklyn, NY, also points to significant under-reporting of sleep problems [114, 115], likely due to unique contextual factors [116, 117]. We surmise decisional conflicts affecting adherence to OSA evaluation must be addressed in the context of sociocultural factors hindering optimal sleep [112, 118, 119]. This study will also provide data to examine effects of contextual factors on sleep behavior of blacks. Identifying contextual factors associated with sleep disturbance and specifically OSA is paramount in designing policies to increase health parity in the United States [120, 121].

Success will be determined by achieving the benchmarks of (1) initial screening of 1256 eligible black participants; (2) acquiring data from 50 enrollees per year in each study arm during years 1–4; (3) acquisition of baseline and 6- and 12-month follow-up outcome data by end of year 4; and (4) dissemination in year 5. We propose to achieve a 15% increase in adoption of OSA care, which would have a significant public health impact.

### Limitations and challenges

Our study has several potential design and analytical limitations and challenges. A primary design challenge is to uphold blinding of participant assignment among key personnel. Only the project coordinator and data manager are aware of participant assignment to either the treatment or intervention group. To safeguard against unblinding, we have set up a very strict policy as to who can have access to data (blinded personnel do not have access to data), how project details are communicated in the team where all data are de-identified, and periodic checks to ensure integrity of data will be made by the data manager, who does not attend research project team meetings nor receives project-related email correspondence. A primary analytical challenge is our use of GEE, which is ideal to test our hypotheses and deal with unmeasured dependencies between outcomes. However, GEE requires a large sample size, which the study is powered for, but if we do not meet adequate sample size our power to make meaningful inferences using GEE will be compromised. As an alternative analytical plan, we would use analysis of covariance, which will improve our ability to detect significant effects on our primary and secondary outcomes using a smaller sample size.

### Trial status

Recruitment for this study began September 2015 and is ongoing.

## Additional files

Additional file 1: Informed consent form. (DOCX 29 kb)

Additional file 2: SPIRIT 2013 Checklist: Recommended items to address in a clinical trial protocol and related documents. (DOCX 63 kb)

## Abbreviations

ABS: Apnea Beliefs Scale
AHI: Apnea-Hypopnea Index
AKT: Apnea Knowledge Test
ARES: Apnea Risk Evaluation System
CPAP: continuous positive airway pressure
CVD: cardiovascular disease
GEE: generalized estimating equation
OSA: obstructive sleep apnea
