# Supplementary material for: Culturally tailored, peer-based sleep health education and social support to increase obstructive sleep apnea assessment and treatment adherence among a community sample of blacks: study protocol for a randomized controlled trial
Source: Trials. 2018 Sep 24;19:519. doi: 10.1186/s13063-018-2835-9 (PMC6154893; doi:10.1186/s13063-018-2835-9)
Supplement: Supplementary file 1 — Informed consent form. (DOCX 29 kb) [file 13063_2018_2835_MOESM1_ESM.docx]

**APPENDICES**

**Research Subject**

**Informed Consent Form**

| **Title of Study:** | Tailored Peer-Based Sleep Health Education and Social Support among Blacks with Obstructive Sleep Apnea  Inset Study Number: s14-01028 |
| --- | --- |
| **Principal Investigator:** | Dr. Girardin Jean-Louis  Department of Population Health  New York University School of Medicine  227 East 30^th^ Street  New York, NY 10016  (646) 501-2623 |
| **Emergency Contact:** | Dr. Azizi Seixas  (646) 501-2672 |

1. **About volunteering for this research study**

You are being invited to take part in a research study. Your participation is voluntary which means you can choose whether or not you want to take part in this study.

People who agree to take part in research studies are called “subjects” or “research subjects”. These words are used throughout this consent form. Before you can make your decision, you will need to know what the study is about, the possible risks and benefits of being in this study, and what you will have to do in this study. You may also decide to discuss this study and this form with your family, friends, or doctor. If you have any questions about the study or about this form, please ask us. If you decide to take part in this study, you must sign this form. We will give you a copy of this form signed by you for you to keep.

1. **What is the purpose of the research study?**

We are asking you to take part in this research study because we want to find out if receiving sleep health education and social support from peers will encourage people to have a home sleep test, and follow-up with treatment recommendations.

Sleep apnea is a sleeping problem in which a person stops breathing at times while he/she sleeps. It is a health problem that commonly affects racial/ethnic minorities. If sleep apnea is untreated it can lead to excessive daytime sleepiness, car accidents, memory problems, heart disease, and increased risk of death.

This research study is being done to collect your thoughts, reactions, and opinions. We will do this through different surveys.

This study is a randomized study. This means, like flipping a coin, if you are eligible, you will have a 50% chance of being assigned to one of two groups. Group A (Intervention group) will receive health education from a trained Peer Health Educator and Group B (Control group) will meet with a salon worker, barbershop worker, or church Health Ministry group. There are no special requirements or criteria to be in either group.

1. **How long will I be in the study? How many other people will be in the study?**

If you are eligible to continue in the study, this study will last 12 months. If you are not eligible, then your participation in the study is over today.

About 1,000 people between the ages of 18 and 90 will be in this study.

This study will be an out-patient study. Your study visits will first be at the site where you were recruited, your home and then follow-up appointments will be at the site where you were recruited, or at our NYU project office.

1. **What will I be asked to do in the study?**

**Subject Participation:**

You cannot participate in this study if you:

• are involved in another sleep study.

• are unable to understand and sign this informed consent form.

• know someone who is participating in this study.

• plan to move away in the next two years.

• had a heart attack or stroke within the past 12 weeks.

• do not identify yourself as a racial/ethnic minority .

• are pregnant.

• are not at risk for sleep apnea.

• refuse to use the WatchPAT^TM^ home sleep test device.

In this study you will be asked complete the following surveys:

- Callahan Cognitive Capacity
- ARES™ Survey
- Sleep Disorders Questionnaire
- SF-12 Health Survey
- Dysfunctional Beliefs and Attitudes about Sleep
- Depression Scale
- Beck Anxiety Inventory
- Apnea Beliefs Scale
- Apnea Knowledge Test
- Functional Outcomes Survey
- Self-Efficacy Scale
- Change Assessment Scale
- Demographics
- Medical History/Clinical Variables
- Epworth Sleepiness Scale
- Process Follow-up Intervention group form
- 7- day Sleep Diary

You will be assigned to Group A (Intervention) or Group B (Control).

Your participation in this study will last for 12 months and will involve up to 8 visits at your barbershop, salon, or church and also in your home. We will schedule them at a time that is convenient for you.

You are free to skip any questions that you prefer not to answer.

At any time in the study, you may decide to withdraw from the study. If you withdraw no more information will be collected from you. When you indicate you wish to withdraw the investigator will ask if the information already collected from you can be used.

If you choose to take part in the study, we will ask you to sign this consent form before you have any procedures with the study staff that are part of the study. If you agree, and sign the consent form, then your first visit will be today.

Below is a list of each phase of the study, including today. This section will help you understand what is expected of you at each visit.

**Phase 1: Screening Visit**

Location: church, community-based event, salon, or barbershop

During this visit, you will be asked to complete surveys to see if you are eligible to take part in the study. The surveys will take about 30-45 minutes to complete. The study coordinator will review the surveys and get back to you by phone within the next week. If you do not meet the requirements, you will be told why.

At this visit, we will:

Ask you about your medical history

Ask you what medications you take – including over-the-counter and prescription medications, vitamins or herbal supplements.

Ask you to complete some forms about your: general health and well-being, quality of life (if you are happy about your life), mental health, emotional health, and mood.

Ask you questions about your sleeping habits.

You will receive $5 to thank you for your time and effort in this study.

**Phase 2**: Enrollment

If you are eligible to continue in this study, and agree to be enrolled, you will then meet with your assigned peer health educator and discuss when are the most convenient times for you to meet.

For the home visit, we will schedule an appointment at your convenience to come to your home to drop off the 7-day Sleep Diary and the WatchPAT^TM^ home sleep test device. The WatchPAT^TM^ home sleep test device will help us to evaluate whether you meet sleep apnea diagnostic criteria. The device is worn on the wrist and fingertip for 7-days. You will be asked to complete the 7-day Sleep Diary including: 1) nap time and duration, 2) medications/drugs promoting night-time sleep or daytime alertness (e.g., caffeine), 3) time to bed, 4) time of lights off, 5) final time of awakening, 6) final time of arising, 7) how many hours you actually sleep, 8) sleep duration, 9) wake after sleep, 10) sleep quality, and 11) daytime sleepiness. At the end of the 7-day period (on the 8^th^ day), we will return to your home to pick up the 7-day Sleep Diary and the WatchPAT^TM^ home sleep test device. On day 8, the technician will also perform the LDX cholesterol and sugar test. They will also administer blood pressure readings for participants. You will receive $50 to thank you for your time and effort in this study.

After the home study is complete, the a board-certified physician associated with the stufy, will then discuss your results with you and make appropriate treatment referrals.

**Phase 3**: Follow-up appointments at 6 and 12 months

Location for 6-month follow-up: church, barbershop, salon or our project office at NYU, or via phone.

Location for 12-month follow-up: over the phone

You will be asked to schedule an appointment to meet with the study coordinator to complete follow-up surveys, which will be similar to those completed at your screening visit. We will also ask if you went to the sleep center, and for what type of visit. If you received a diagnosis for sleep apnea and received treatment, we will request your sleep test results and treatment information. At the 6-month follow-up, you will receive $30 to thank you for your time and effort in this study. For the 12-month follow-up, the research staff will call you and administer a few surveys over the phone, which will take about 5 minutes or less.

1. **What are the possible risks or discomforts?**

If you are at high risk for sleep apnea, you will be referred to go for a sleep test. Sensors used for the sleep study recordings may cause minor skin irritation among people with sensitive skin; this does not usually require medical attention. Such irritation can easily be resolved with over-the-counter ointment that can be obtained at a local drug store. All safety measures will be taken to prevent any harm. Also, there may be some questions which may cause anxiety or discomfort, but you do not have to answer them.

As a participant, there is a risk to your confidentiality but your personal information will be removed from all our databases and your survey will be kept in a locked cabinet. Also, your surveys will have an ID code that we will use to identify you. No one outside of the study team will have access to this information.

1. **What are the possible benefits of the study?**

We cannot promise that you will benefit from being in this study. However, possible benefits include learning about general sleep health, specifically sleep apnea, and receiving treatment for sleep apnea. We do hope that in the future, people might benefit from this study because the information that we learn from this study will be used to raise awareness of sleep apnea and improve the health of our community.

The alternative to being in this study is not to participate. You are eligible for Group A (Intervention) or Group B (Control) because you may have sleep apnea.

1. **What other choices do I have if I do not participate?**

You may choose to not participate in this research study.

1. **Will I be paid for being in this study?**

You will be paid to take part in this study. We will pay you by cash.

You will be paid per completed visit. You will receive $5 for your screening visit; $60 for the baseline and home study visit; and $30 for the 6-month follow-up visit, and $30 for the 12-month follow-up visit. If you choose to leave or are withdrawn from the study for any reason before finishing the entire study*,* you will be paid dependent on the point of withdrawal. If you complete all the study visits, you will receive $125 for being in this study.

1. **Will I have to pay for anything?**

There will be no costs to you for being in this study.

NIH is providing financial support to NYU School of Medicine to conduct this study.

1. **What happens if I am injured from being in the study?**

If you sustain any injury during the course of the research or experience any side effect to a study treatment, please contact the Principal Investigator Dr. Jean-Louis at the following telephone number 646-501-2591. If such complications arise, the study doctor will assist you in obtaining appropriate medical treatment but this study does not provide financial assistance for medical or other injury-related costs. You do not give up any rights to seek payment for personal injury by signing this form.

1. **When is the study over? Can I leave the study before it ends?**

It is your choice to be in this study. No one can force you to be in the study. No one can force you to stay in the study. You can leave the study at any time. Leaving the study will not affect the care you receive.

The study doctor, the sponsor of the study or government monitors like the FDA or NYU School of Medicine IRB can take you out of the study without your permission at any time for the following reasons:

you do not follow the study doctor’s instructions

the study staff finds out you do not meet the requirements of the study

the study is stopped

taking part in the study becomes harmful to your health

If you choose to leave the study or if you are taken out of the study, you may be asked to come for a final visit to have some follow-up study evaluations. These are for your safety and protection. Please note all the data that has been collected on you to the point that you leave the study will remain part of the study.

You may also withdraw your Authorization for us to use or disclose your protected health information for the study.

If you do decide to withdraw your consent, we ask that you contact Dr. Jean-Louis and let him know that you are withdrawing from the study. His mailing address is NYU Langone Medical Center, 227 East 30th Street, 6^th^ fl, New York, NY 10016

1. **How will my information be protected?**

NYU Langone Medical Center, which includes NYU Hospitals Center and NYU School of Medicine, is committed to protecting the privacy and confidentiality of your health information. We are asking for your permission to use and to disclose your health information in connection with this study. You have the right not to give us this permission, in which case you will not be able to participate in this study. If you do not give this permission, your treatment outside of this study, payment for your health care, and your health care benefits will not be affected.

**What information about me may be used or shared with others?**

The following information may be used or shared in connection with this research:

- Information in your medical record and research record, for example, results from your physical examinations, laboratory tests, procedures, questionnaires and diaries.

You have a right to access information in your medical record. In some cases when necessary to protect the integrity of the research, you will not be allowed to see or copy certain information relating to the study while the study is in progress, but you will have the right to see and copy the information once the study is over in accordance with NYU Langone Medical Center policies and applicable law.

**Why is my information being used?**

Your health information will be used by the research team and others involved in the study to conduct and oversee the study.

**Who may use and share information about me?**

The following individuals may use, share or receive your information for this research study:

- The Principal Investigator, study coordinators, other members of the research team, and personnel responsible for the support or oversight of the study.
- The study sponsor: NIH
- Governmental agencies responsible for research oversight (e.g., the Food and Drug Administration or FDA).
- Health care providers who provide services to you in connection with this study, and laboratories or other individuals who analyze your health information in connection with this study.
- Other study sites
  - Contract research organizations
  - Central research laboratories
  - Study related committees/boards/centers (Data & Safety Monitoring Board, Endpoint Committees, Clinical or Data Coordination Centers, etc.)

Your information may be re-disclosed or used for other purposes if the person who receives your information is not required by law to protect the privacy of the information.

**How long may my information be used or shared?**

Your permission to use or share your personal health information for this study will never expire unless you withdraw it.

**Can I change my mind and withdraw permission to use or share my information?**

Yes, you may withdraw or take back your permission to use and share your health information at any time. If you withdraw your permission, we will not be able to take back information that has already been used or shared with others. To withdraw your permission, send a written notice to the principal investigator for the study noted at the top of page 1 of this form. If you withdraw your permission, you will not be able to stay in this study.

1. **Optional permission for future use**

NYULMC would also like to store, use, and share your health information from this study in research databases or registries for future research conducted by NYULMC or its research partners. Such health information may include biological samples from the study. To give this additional permission, check the box below and write your initials where indicated. You may still participate in this study even if you do not give us this additional permission.

NYULMC will continue to protect the confidentiality and privacy of this information as required by law and our institutional polices. If you give this additional permission, you will continue to have the rights described in this form. You have the right to take back this additional permission at any time.

| Checking this box indicates my permission to store, use, and share my health information from this study in research databases or registries for future research conducted by NYULMC or its research partners. | \|  \| \| --- \|   Subject Initials |
| --- | --- | --- |
|  |  |

1. **Permission to contact you about future studies on sleep apnea:**

I authorize the principal investigator and his or her co-investigators to contact me about future research on Obstructive Sleep Apnea within the Medicine Department provided that this future research is approved by the original IRB of record and that the principal investigator and co-investigator are affiliated with the research protocol.

If I agree, then someone from Dr. Jean-Louis’s research staff might contact me in the future and he or she will tell me about a research study. At that time, I can decide whether or not I am interested in participating in a particular study. I will then have the opportunity to contact the researcher to schedule an appointment to be fully informed about the research project.

I agree to be contacted by the Principal Investigator or Co-Investigators.

I **do not** want to be contacted by the Principal Investigator or Co-Investigators.

__________________________________________________________________

Signature of participant or legal representative Date

Your permission to allow us to contact you about future research would be greatly appreciated, but it is completely voluntary. If you choose not to allow us to contact you, it will not affect your care at any of the NYUSM facilities. Please understand that giving your permission to do this is only for the purpose of helping us identify subjects who may qualify for one of our future research studies. It does not mean that you must join in any study.

1. **The Institutional Review Board (IRB) and how it protects you**

**What is the Institutional Review Board (IRB) and how does it protect me?**

The IRB reviews all human research studies – like the one you are considering. The IRB protects the rights and welfare of the people taking part in the research studies. The IRB follows rules and guidelines from the Federal Government and reviews each research study using these guidelines. The IRB also reviews research to make sure the risks for all studies are as small as possible.

The NYU School of Medicine’s IRB is made up of:

Doctors

Nurses

Non-scientists

People from the Community

You may contact the IRB if you have any questions about your rights as a subject, if you think you are not treated fairly or if you have any questions about this research study. The NYU IRB Office number is (212) 263-4110.

1. **Who can I call with questions, or if I’m concerned about my rights as a research subject?**

If you have questions, concerns or complaints regarding your participation in this research study or if you have any questions about your rights as a research subject, you should speak with the Principal Investigator listed on top of the page 1 of this consent form. If a member of the research team cannot be reached or you want to talk to someone other than those working on the study, you may contact the Institutional Review Board (IRB) at (212) 263-4110.

| **When you sign this form**, you are agreeing to take part in this research study as described to you. This means that you have read the consent form, your questions have been answered, and you have decided to volunteer. |
| --- |

|  |  |  |  |  |
| --- | --- | --- | --- | --- |
| Name of Subject (Print) |  | Signature of Subject |  | Date |
|  |  |  |  |  |
| Name of Person Obtaining Consent (Print) |  | Signature of Person Obtaining Consent |  | Date |
